# Spinocerebellar ataxia 27B: A novel, frequent and potentially treatable ataxia

**DOI:** 10.1002/ctm2.1504

**Published:** 2024-01-27

**Authors:** David Pellerin, Matt C. Danzi, Mathilde Renaud, Henry Houlden, Matthis Synofzik, Stephan Zuchner, Bernard Brais

**Affiliations:** ^1^ Department of Neurology and Neurosurgery, Montreal Neurological Hospital and Institute McGill University Montreal Quebec Canada; ^2^ Department of Neuromuscular Diseases, UCL Queen Square Institute of Neurology and The National Hospital for Neurology and Neurosurgery University College London London UK; ^3^ Dr. John T. Macdonald Foundation Department of Human Genetics and John P. Hussman Institute for Human Genomics University of Miami Miller School of Medicine Miami Florida USA; ^4^ INSERM‐U1256 NGERE Université de Lorraine Nancy France; ^5^ Service de Neurologie, CHRU de Nancy Nancy France; ^6^ Service de Génétique Clinique, CHRU de Nancy Nancy France; ^7^ Division of Translational Genomics of Neurodegenerative Diseases Hertie‐Institute for Clinical Brain Research and Center of Neurology, University of Tübingen Tübingen Germany; ^8^ German Center for Neurodegenerative Diseases (DZNE) Tübingen Germany; ^9^ Department of Human Genetics McGill University Montreal Quebec Canada

**Keywords:** 4‐aminopyridine, cerebellar ataxia, FGF14, GAA‐FGF14 ataxia, genetics, repeat expansion disorder, therapy

## Abstract

Hereditary ataxias, especially when presenting sporadically in adulthood, present a particular diagnostic challenge owing to their great clinical and genetic heterogeneity. Currently, up to 75% of such patients remain without a genetic diagnosis. In an era of emerging disease‐modifying gene‐stratified therapies, the identification of causative alleles has become increasingly important. Over the past few years, the implementation of advanced bioinformatics tools and long‐read sequencing has allowed the identification of a number of novel repeat expansion disorders, such as the recently described spinocerebellar ataxia 27B (SCA27B) caused by a (GAA)•(TTC) repeat expansion in intron 1 of the fibroblast growth factor 14 (*FGF14*) gene. SCA27B is rapidly gaining recognition as one of the most common forms of adult‐onset hereditary ataxia, with several studies showing that it accounts for a substantial number (9–61%) of previously undiagnosed cases from different cohorts. First natural history studies and multiple reports have already outlined the progression and core phenotype of this novel disease, which consists of a late‐onset slowly progressive pan‐cerebellar syndrome that is frequently associated with cerebellar oculomotor signs, such as downbeat nystagmus, and episodic symptoms. Furthermore, preliminary studies in patients with SCA27B have shown promising symptomatic benefits of 4‐aminopyridine, an already marketed drug. This review describes the current knowledge of the genetic and molecular basis, epidemiology, clinical features and prospective treatment strategies in SCA27B.

## Graphical abstract

Spinocerebellar ataxia 27B is caused by a (GAA)•(TTC) repeat expansion in intron 1 of *FGF14* and is a common cause of adult‐onset ataxia, accounting for 9–61% of previously undiagnosed cases in different ethnically diverse cohorts.

The core phenotype consists of a late‐onset slowly progressive pan‐cerebellar syndrome with frequent oculomotor signs.

Episodic symptoms, visual disturbances and downbeat nystagmus are commonly observed in patients with SCA27B.

4‐Aminopyridine is a promising symptomatic treatment.

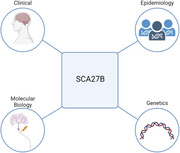

## 1 INTRODUCTION

Despite the recent progress in the rates of diagnosis of rare diseases brought forward by the advent of next‐generation sequencing, more than half of patients with a suspected neurogenetic disorder remain without a molecular diagnosis.^1^, ^2^, ^3^, ^4^ The diagnostic gap is further widened in hereditary cerebellar ataxia, a group of highly clinically and genetically heterogeneous disorders manifesting with progressive cerebellar dysfunction,^5^ and in particular in patients with sporadic late‐onset ataxia.^1^, ^6^, ^7^, ^8^, ^9^, ^10^ Diagnostic yields still remain below 35% in patients with hereditary ataxia undergoing whole‐genome sequencing.^1^ This is thought to be in part due to the technical limitations of standard short‐read sequencing analysis to identify complex sequence variations, such as tandem repeat expansions,^11^, ^12^ that are likely to hide in non‐coding regions of the genome.^13^ In an era of emerging disease‐modifying gene‐stratified therapies, the identification of causative alleles in neurodegenerative ataxia has become increasingly important.^14^, ^15^, ^16^

The recent development of advanced bioinformatics tools and long‐read sequencing technologies are predicted to close the diagnostic gap in hereditary ataxia by fostering the identification of previously inaccessible genomic variations.^17^, ^18^ Their early implementation has already changed the genetic landscape of late‐onset ataxias by enabling the identification of intronic non‐reference pentanucleotide repeat expansions in the replication factor C subunit 1 (*RFC1*) gene^19^, ^20^ and, more recently, (GAA)•(TTC) repeat expansions in intron 1 of the fibroblast growth factor 14 (*FGF14*) gene.^21^, ^22^ These non‐coding repeat expansions, which respectively cause the cerebellar ataxia, neuropathy and vestibular areflexia syndrome (CANVAS, MIM 614575) and spinocerebellar ataxia (SCA) 27B (SCA27B/GAA‐*FGF14* ataxia, MIM 620174), account for a substantial share of previously undiagnosed cases of sporadic and familial late‐onset ataxia. SCA27B is increasingly being recognised as one of the most common causes of autosomal dominant ataxia in the European population.^23^ In this review, we will discuss the genetics and molecular basis of the recently described SCA27B, its epidemiology, clinical features and prospective treatment strategies.

## 2 GENETIC BASIS OF SCA27B

**FIGURE 1:**
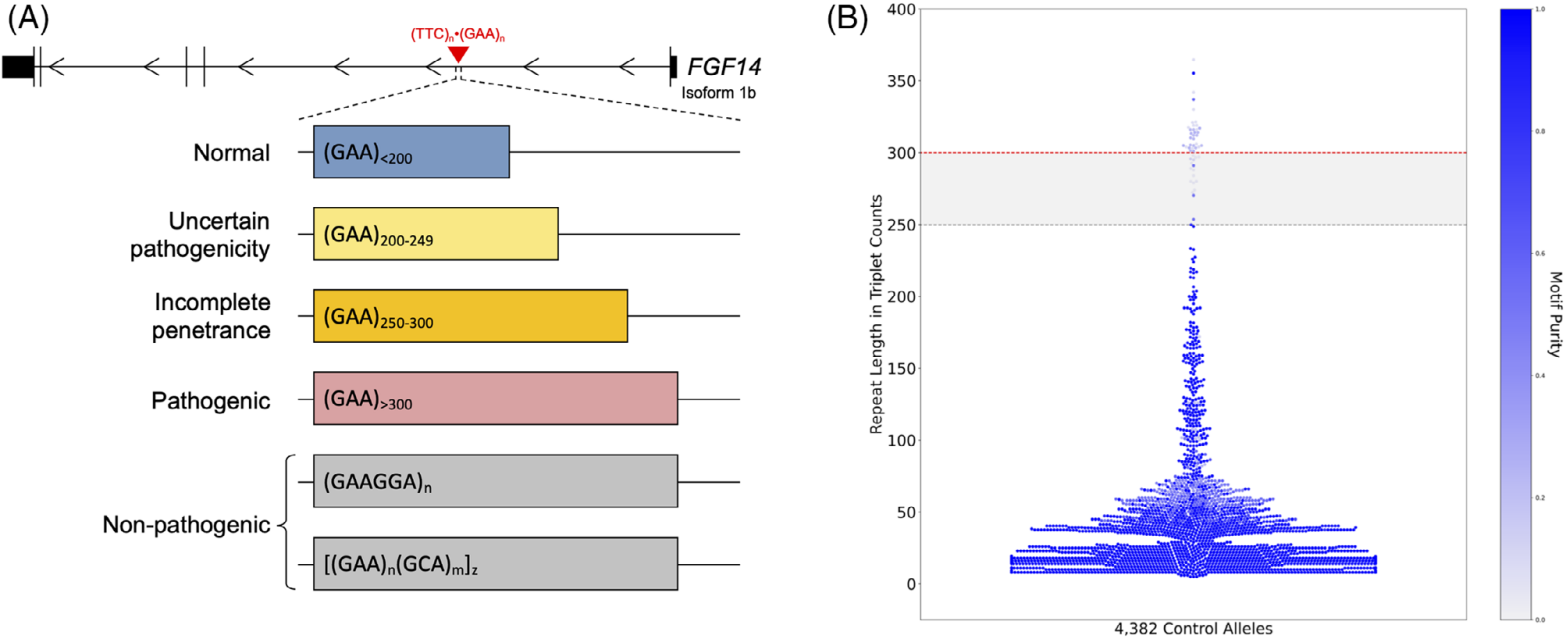
Length and motif polymorphism of the *FGF14*‐SCA27B repeat locus. (A) Diagram of the *FGF14* gene, isoform 1b showing the location of the (GAA)_n_•(TTC)_n_ repeat locus in the first intron (GRCh38, chr13:102,161,575‐102,161,726) with representation of the normal alleles, alleles of uncertain pathogenicity, incompletely penetrant alleles, pathogenic alleles and likely non‐pathogenic non‐GAA‐pure alleles, such as (GAAGGA)n*_n_* and [(GAA)*_n_* (GCA)*_m_*]*_z_*. (B) Swarm plot showing the allele distribution of the *FGF14* repeat locus in 2191 non‐ataxic controls (4382 chromosomes) as assessed by PacBio HiFi long‐read sequencing.^32^ The colour of the data points is a function of the GAA repeat motif purity, with dark blue indicating pure and lighter blue impure/interrupted motif (a hue scale is shown on the right *y* axis). The dashed grey line and the shaded grey area indicate the incompletely penetrant range of (GAA)_250–300_ repeat units, and the dashed red line represents the pathogenic threshold of (GAA)_>300_ repeat units. Two non‐GAA‐pure alleles of over 800 repeat units were excluded for clarity.

Two independent groups simultaneously reported the genetic basis of SCA27B.^21^, ^22^ Pellerin et al. identified expanded GAA tracts in the first intron of the *FGF14* gene (GRCh38, chr13:102,161,575‐102,161,726) that were associated with late‐onset cerebellar ataxia in 128 patients. Rafehi et al. showed similar results in 28 patients. Missense, nonsense and frameshift variations in *FGF14* had previously been associated with SCA27A,^24^, ^25^, ^26^ and *Fgf14* knock out mice had previously been shown to exhibit an ataxic phenotype.^27^ The *FGF14*‐SCA27B repeat locus was found to have a high degree of length and motif polymorphism among controls (Figures 1A and B). Both groups suggested that expansions greater than 250 GAA repeat units were pathogenic, albeit with reduced penetrance for (GAA)_250–299_ alleles (Figure 1A).^21^, ^22^ This 250 GAA repeat unit threshold was based on familial segregation data from several French‐Canadian families with SCA27B.^21^ The two publications found that expansions above 300 GAA repeat units appear fully penetrant based on the largest GAA‐pure alleles observed in 408 and 311 controls, respectively.^21^, ^22^ Biallelic expansions have also been identified in a few patients.^21^, ^28^, ^29^, ^30^ It is likely that the incomplete and fully penetrant thresholds will need to be refined as more patient and control screening is completed. In fact, two recent studies have shown evidence that alleles as small as 200 GAA repeat units might be pathogenic.^23^, ^31^ The first demonstrated segregation with disease of alleles of 234 GAA repeat units and greater in a family with late‐onset slowly progressive ataxia.^23^ The second study showed enrichment of alleles with 200–249 GAA repeat units among individuals with downbeat nystagmus (DBN) syndromes relative to controls.^31^ The phenotype of the patients with 200–249 GAA repeat units did not significantly differ from those with 250 or more GAA repeat units.

The high degree of motif variation observed at this locus further complicates efforts to identify the pathogenic threshold. Long‐read sequencing of close to 2200 controls has revealed a wide variety of alternative motifs present at the SCA27B locus, particularly at longer allele lengths.^32^ Many of these alternative motifs have GAA interleaved with other triplets at regular intervals, such as [(GAA)_4_(GCA)_1_]*_n_*. In fact, 88.9% of alleles longer than 250 repeat units in controls subjected to long‐read sequencing were identified to be GAA‐impure (Figure 1B).^32^ To our knowledge, no studies have yet shown segregation of non‐GAA repeats with SCA27B.^21^, ^23^, ^33^, ^34^

The SCA27B locus also exhibits high levels of intergenerational instability, dependent on the length and GAA purity of the tandem repeat.^21^, ^32^ GAA‐pure tracts longer than 75 repeat units most often expand upon maternal transmission and contract upon paternal transmission, with the degree of instability increasing proportional to GAA tract length.^21^, ^32^ The significant instability of the *FGF14* GAA repeat locus upon intergenerational transmission likely explains in part the high incidence of sporadic cases of SCA27B, varying between 15 and 50%.^21^, ^30^, ^35^ Furthermore, contraction of the size of the GAA repeat on paternal transmission may lead to transmission of normal or incompletely penetrant alleles to the offspring, resulting in ‘generation skipping’ of the disease.^21^ This differential transmission dynamic also likely accounts for the reduced male transmission of the disease.^21^, ^31^ In contrast, non‐GAA pure tracts are largely stable upon intergenerational transmission.^32^ In comparison, GAA‐pure tracts with fewer than 30 repeat units, which represent the most common set of alleles in the general population, are stable upon intergenerational transmission.^32^ These short, pure, stable alleles invariably contain a particular 17‐bp deletion‐insertion on their 5′ flank which longer sequences do not.^32^ Whether there is a causal relationship between this flanking variant and intergenerational transmission stability is under investigation.

## 3 EPIDEMIOLOGY AND REGIONAL DISTRIBUTION

Population‐based studies and epidemiological surveys have revealed prevalence rates ranging from 1.5 to 4.0 per 100 000 population worldwide for SCAs^36^ and from 2.2 to 12.4 per 100 000 population for so‐called sporadic adult‐onset ataxia of unknown aetiology.^37^, ^38^, ^39^, ^40^ While the prevalence of SCA27B is difficult to estimate at this time, a recent single‐centre systematic comparative study of 320 consecutive patients with SCA from Germany showed that SCA27B has a frequency (16%) in the same range as the common polyglutamine SCA subtypes SCA3 (19%), SCA1 (12%), SCA6 (12%) and SCA2 (9%).^23^ This observation is in line with other studies that have shown SCA27B to have a frequency of approximately 15−30% in European cohorts of patients with unsolved adult‐onset ataxia.^21^, ^22^, ^30^, ^35^, ^41^, ^42^ Such high prevalence may be reflective of a population structure in individuals of European descent who appear to more commonly carry larger *FGF14* GAA alleles compared to other populations.^21^, ^22^ Future studies are needed to define the regional prevalence of SCA27B, although ongoing screening efforts suggest that SCA27B is also a relatively common cause of late‐onset ataxia in South Asia (10%)^21^ and Brazil (9%).^43^ However, SCA27B may not be as common in East Asian populations, as it was not identified in 312 patients with suspected spinocerebellar degeneration of unknown cause from the Hokkaido island in northern Japan in one study^44^ and was identified in only 11 of 940 individuals (1.2%) from Japan with chronic progressive cerebellar ataxia in another study.^45^

In Quebec, a frequency of SCA27B as high as 60% has been reported among French‐Canadian patients with previously unexplained adult‐onset ataxia, making it the most common genetic cause of adult‐onset ataxia in this population.^21^, ^46^ The observation of a common disease haplotype shared by some French‐Canadian patients suggest that the high proportion of SCA27B in this population may correspond to a founder effect in this population known to be enriched for such effects.^47^ Similarly, a previous study identified that three Australian patients shared a disease haplotype.^22^ Whether the *FGF14* GAA repeat expansion arose from a common haplotype is yet to be determined, although the finding of SCA27B in patients of different ancestries suggests that this expansion is not limited to a single ancestral haplotype. This is further supported by the recent observation that 54% of patients with SCA27B did not carry the rs72665334 C > T single nucleotide variant,^31^ which was part of the disease haplotype shared by the three Australian patients.^22^

## 4 PHENOTYPIC PROFILE AND DISEASE PROGRESSION

Table 1 summarises the main phenotypic features of SCA27B.

**TABLE 1:** Frequency of select phenotypic features of spinocerebellar ataxia 27B.

Feature	Frequency (range)	Comment	References
Gait ataxia	95–100%		^21^, ^22^, ^30^, ^35^, ^41^, ^42^, ^50^, ^51^
Upper limb ataxia	44–71%		^22^, ^30^, ^35^, ^50^
Episodic symptoms	13–80%	May be triggered by alcohol intake, physical activity, or caffeine	^21^, ^30^, ^31^, ^35^, ^41^, ^50^, ^51^
Cerebellar dysarthria	12–74%	Rarely severe	^21^, ^22^, ^30^, ^35^, ^41^, ^42^, ^50^, ^51^
Cerebellar oculomotor signs	80–96%	Includes saccadic pursuit, dysmetric saccades, rebound nystagmus, gaze‐evoked nystagmus, downbeat nystagmus, impaired visual fixation suppression of the vestibulo‐ocular reflex	^22^, ^30^, ^42^, ^50^
Downbeat nystagmus	10–67%	May be episodic and/or occur in isolation with other cerebellar oculomotor signs at disease onset	^21^, ^35^, ^50^, ^51^
Diplopia, oscillopsia, visual blurring	40–68%		^21^, ^30^, ^41^, ^42^, ^50^, ^51^
Vertigo and/or dizziness	21–67%		^21^, ^30^, ^35^, ^41^, ^42^, ^50^, ^51^
Postural tremor of upper limbs	10–27%		^21^, ^35^, ^50^
Bilateral vestibulopathy	10–75%	Vestibular function remains mildly impaired	^22^, ^30^, ^31^, ^35^, ^50^
Cerebellar atrophy on brain MRI	60–97%	Cerebellar vermis > hemisphere; remains mild to moderate despite prolonged disease duration	^21^, ^30^, ^35^, ^42^, ^50^

Summary of the key phenotypic features of spinocerebellar ataxia 27B (SCA27B). The range of the frequencies for each feature as reported in the various series published to date is shown.

In comparison with the common polyglutamine SCAs which typically have a disease onset in the third to fifth decade,^48^, ^49^ SCA27B consistently presents in the fifth to seventh decade.^21^, ^22^, ^23^, ^30^, ^31^, ^41^, ^42^, ^50^ Some – but not all – cases carrying biallelic *FGF14* GAA repeat expansions may manifest before the age of 30 and/or have a more severe phenotype.^28^, ^29^ The GAA repeat length only appears to correlate weakly with the age at onset,^21^, ^22^, ^30^, ^31^, ^42^ in contrast to polyglutamine SCAs.^49^ Several factors may account for such weak correlation in SCA27B, including patients’ failure to recognise early episodic symptoms, co‐occurrence of ‘second‐hit’ neurological disease and medical comorbidities,^30^, ^31^ unknown genetic modifiers and possibly brain somatic mosaicism.

The core phenotype of SCA27B consists of a slowly progressive pancerebellar syndrome predominantly characterised by gait ataxia and cerebellar oculomotor impairment.^21^, ^30^, ^31^, ^42^ The severity of the cerebellar involvement appears to follow a caudal‐to‐rostral gradient, as suggested by the greater impairment of gait, stance and lower extremities compared to upper extremities and speech in patients with SCA27B.^30^ One study found no association between disease severity or progression and the length of the repeat expansion.^30^ While the majority of patients present with gait unsteadiness at disease onset, almost half of patients report episodic symptoms such as vertigo and/or dizziness, visual disturbances (diplopia, oscillopsia, blurring) and dysarthria.^21^, ^30^, ^50^ The frequency and duration of episodes of ataxia are highly variable and may last from minutes to days and occur daily to monthly, respectively. Episodic symptoms, which may antedate permanent ataxia by several years,^21^, ^31^, ^41^ may fail to be recognised by patients and providers alike to be related to an underlying cerebellar disorder. This may partly account for the significant discrepancy in the frequency of episodic symptoms reported in studies published thus far – varying from 13 to 80%^21^, ^30^, ^41^, ^50^, ^51^ – and highlights the need to establish an accepted clinical definition of these symptoms. We had previously proposed the following definition: ‘a recognizable constellation of symptoms, which are recurrent, intermittent and discrete with clear onset and offset from the patient's established baseline, and can appear unprovoked or be induced, for example, by alcohol or physical activity; patients must have episodic cerebellar symptoms (gait ataxia, dysarthria, diplopia, oscillopsia, vertigo and/or dizziness or appendicular ataxia), but can also have other episodic symptoms’.^51^ In addition to episodic cerebellar symptoms, patients may rarely experience episodic ‘brain fog’^52^ or sudden falls/‘drop attacks’.^51^ More than half of patients with SCA27B display sensitivity to alcohol, which may trigger attacks of ataxia or dramatically worsen baseline ataxia.^41^ Physical exertion has similarly been noted to be a common trigger for episodic symptoms.^21^ Caffeine intake may also occasionally trigger episodes of ataxia.^51^

DBN, cerebellar oculomotor signs, impaired visual fixation suppression of the vestibulo‐ocular reflex (VOR), vertiginous symptoms and visual disturbances frequently co‐occur at disease onset, likely reflecting early preferential involvement of the cerebellar flocculus and paraflocculus in SCA27B.^31^ DBN is observed in up to 70% of patients with SCA27B and, as such, appears to be a particularly specific clinical manifestation of SCA27B in late‐onset ataxia,^21^, ^50^ although not exclusive as it is also a frequent feature of SCA6.^53^ Further supporting the strong association between SCA27B and DBN, a recent study showed that *FGF14* GAA repeat expansions are a remarkably frequent genetic cause of DBN syndromes, accounting for almost 50% of hitherto idiopathic cases, especially when associated with additional cerebellar oculomotor signs.^31^ Patients with DBN carrying an *FGF14* repeat expansion all exhibited additional cerebellar oculomotor signs (saccadic pursuit, dysmetric saccades, gaze‐evoked nystagmus, rebound nystagmus, impaired visual fixation suppression of the VOR), although only 43% displayed cerebellar ataxia despite protracted disease evolution. This finding suggests that the disease may remain limited to the cerebellar oculomotor system without broader cerebellar involvement in a subset of patients carrying an *FGF14* GAA repeat expansion, raising the possibility that cerebellar ataxia – the defining feature of SCA27B – is not a universal feature of GAA‐*FGF14*‐related disease. Future natural history studies will be needed to confirm this initial observation, which, however, may indicate that the true prevalence of GAA‐*FGF14*‐related disease may be higher than reported. Furthermore, visual disturbances – including diplopia – and vertigo, both relatively uncommon in polyglutamine SCAs,^54^ are observed in 40−68 and 21−67% of patients with SCA27B, respectively.^21^, ^30^, ^42^ Together, episodic symptoms, cerebellar floccular/parafloccular oculomotor signs (in particular DBN), visual disturbances and vertigo appear to be particularly common clinical features of SCA27B.^21^, ^30^, ^31^, ^42^, ^50^

In addition to cerebellar impairment, vestibular hypofunction and afferent sensory defect are commonly noted in SCA27B.^30^, ^50^ Bilateral vestibulopathy (BVP) is noted in 10–75% of patients with SCA27B and appears to develop later in the disease course and to remain relatively mild (not evolving into frank vestibular areflexia).^22^, ^30^, ^31^, ^55^ While initial series suggested that polyneuropathy is not a core feature of SCA27B,^21^, ^22^ subsequent studies have shown that some patients may develop mild axonal sensory or sensorimotor polyneuropathy.^42^, ^50^ Whether the polyneuropathy is pathophysiologically related to SCA27B or simply reflective of an age‐related disease process remains to be established. However, the presence of sensorimotor neuropathy may potentially serve as a useful feature to distinguish SCA27B from *RFC1*‐related CANVAS and disease spectrum,^50^ in which motor neuropathy is most commonly minimal or absent.^56^, ^57^, ^58^ In addition, while sensory neuronopathy is not part of the phenotypic spectrum of SCA27B, it is a hallmark of *RFC1*‐related disease^56^; indeed, the diagnosis of *RFC1*‐related disease is highly unlikely in presence of isolated cerebellar ataxia without sensory neuronopathy.^59^ Even in absence of polyneuropathy proven on electrodiagnostic studies, a substantial proportion of patients with SCA27B (∼50%) exhibit reduced distal vibration sense, suggesting a dysfunction of the afferent tracts.^30^ In contrast to the common multisystemic polyglutamine SCAs^48^ and many other genetic ataxias,^60^ SCA27B does not frequently affect lower motor neurons and the pyramidal and extrapyramidal systems.^21^, ^22^, ^30^, ^42^, ^50^ Autonomic dysfunction, mainly manifesting as urinary urgency, is observed in 20−30% of patients later in the disease course and tends to remain mild, not evolving into frank autonomic failure.^30^, ^42^ The limited multisystemic involvement in SCA27B was reflected in one study by the overall low burden of non‐ataxia features measured by the Inventory of Non‐Ataxia Signs scale, which increased only slowly with disease duration.^30^

Disease progression in SCA27B, averaging 0.23–0.40 SARA point per year,^30^, ^42^ is considerably slower than in other common late‐onset genetic ataxias, such as SCA6 (0.80 SARA point/year)^48^ and *RFC1*‐related disease (1.30 SARA points/year).^58^ The slow disease progression in SCA27B is also reflected in the gradual accrual of functional disability, amounting to only 0.10 FARS stage per year of disease duration.^31^ Correspondingly, half of patients require unilateral walking aid after 8 years and bilateral walking aids after 15 years of disease duration, respectively.^30^ In comparison, the use of wheelchair remains uncommon even after protracted disease duration.

Magnetic resonance imaging of the brain shows cerebellar atrophy, most pronounced in the vermis, in 60–100% of patients.^21^, ^30^, ^42^ Cerebellar atrophy tends to remain mild to moderate despite prolonged disease duration. The pattern of atrophy, which affects the vermis more than the hemispheres, corresponds well to the observed clinical syndrome mainly affecting balance and stance.^30^ In comparison, brainstem atrophy does not appear to be a feature of SCA27B.^30^

## 5 DIFFERENTIAL DIAGNOSIS OF SCA27B

The differential diagnosis of SCA27B is broad and encompasses acquired, hereditary and neurodegenerative causes of adult‐onset ataxias.^61^ Important differential diagnoses to consider include multiple system atrophy, cerebellar type (MSA‐c), *RFC1*‐related CANVAS and disease spectrum, SCA5, SCA6, SCA8, episodic ataxia type 2 (EA2) and fragile X‐associated tremor/ataxia syndrome.

Compared to SCA27B, MSA‐c is a fatal, sporadic, rapidly progressive adult‐onset neurodegenerative disorder mainly characterised by autonomic failure, poorly levodopa‐responsive parkinsonism and cerebellar ataxia.^62^, ^63^ Approximately 60% of patients with MSA become wheelchair‐bound after 5 years and the mean survival is 6–10 years from symptom onset.^62^, ^63^ Multisystem involvement seen in MSA‐c, including extrapyramidal features, pyramidal features, rapid eye movement sleep behaviour disorder, significant dysphagia and autonomic failure, is not characteristic of SCA27B. The absence of episodic symptoms and the presence of putamen, middle cerebellar peduncles (MCPs) and pons atrophy, as well as cruciform T2‐weighed hyperintensity in the pons and increased diffusivity of the putamen and MCPs on MRI^64^ may help differentiate MSA‐c from SCA27B.

*RFC1*‐related disease and SCA27B have been shown to have partial phenotypic overlap, based on the observation that both disorders may present with cerebellar ataxia, neuropathy and BVP.^21^, ^22^, ^50^ While sensory neuronopathy^56^ and chronic cough^58^ are highly prevalent features in *RFC1*‐related disease, they are uncommon in SCA27B.^50^, ^55^ Furthermore, episodic ataxia is not a feature of *RFC1*‐related disease, in contrast to SCA27B. The vestibular function appears to be significantly more impaired in *RFC1*‐related disease compared to SCA27B.^55^ Finally, the pattern of inheritance (autosomal recessive in *RFC1*‐related disorder and autosomal dominant in SCA27B) may also help differentiating both conditions, although patients with SCA27B may present sporadically or with seemingly recessive inheritance.^21^, ^50^

SCA6 and EA2 have significant phenotypic overlap with SCA27B. Like patients with SCA27B, patients with SCA6 present with slowly progressive, adult‐onset pure cerebellar ataxia and oculomotor signs, including DBN in a substantial number of cases.^65^, ^66^ The mean age at onset of patients with SCA6 is approximately 5−10 years earlier (mean age, 49−55 years)^48^, ^49^, ^66^ than that of patients with SCA27B (mean age, 55−67 years).^21^, ^22^, ^23^, ^30^, ^31^, ^35^, ^42^, ^51^ Similar to SCA27B, vertigo and visual disturbances, such as diplopia and oscillopsia, are common features of SCA6.^65^, ^66^, ^67^ However, episodic ataxia is less frequent and cerebellar dysarthria more frequent in SCA6 compared to SCA27B, respectively occurring in less than 15% and more than 90% of patients with SCA6.^65^, ^66^, ^67^ Furthermore, positional DBN triggered by the head‐down position is more common than spontaneous DBN in SCA6,^53^, ^66^ whereas spontaneous DBN is characteristic of SCA27B.^21^, ^31^, ^68^ Pyramidal tract signs, peripheral neuropathy and dystonia, which are uncommon in SCA27B, may occur in up to 50, 40 and 25% of patients with SCA6, respectively.^65^ Finally, like SCA27B, brain MRI in SCA6 shows isolated cerebellar atrophy, predominant in the vermis.^69^

In comparison, EA2 typically develops in childhood or early adolescence, although rare patients manifesting in adulthood have been described.^70^, ^71^, ^72^ Like patients with SCA27B, patients with EA2 initially experience episodic ataxia with paroxysmal attacks of ataxia, dysarthria, diplopia and vertigo. Paroxysmal attacks of ataxia in EA2 may also be associated with dystonia, hemiplegia and tonic upward gaze, which are not observed in SCA27B. Episodes of ataxia, which may be triggered by exercise, emotional stress and alcohol, may last for hours and inter‐ictal gaze‐evoked nystagmus and DBN are frequently observed.^71^, ^73^, ^74^ Inter‐ictal progressive cerebellar ataxia and cerebellar atrophy on MRI may eventually develop in some patients with EA2.^71^, ^72^, ^75^ Acetazolamide also appears to be more effective in EA2 than in SCA27B to decrease the frequency and severity of episodes of ataxia.^51^, ^76^

## 6 NEUROPATHOLOGICAL FINDINGS

The neuropathological findings of SCA27B have so far been described in three patients and appear to be restricted to the cerebellum.^21^, ^30^ All cases displayed cerebellar cortical atrophy that was more prominent in the vermis than the hemispheres, consistent with the pattern of atrophy observed on MRI. Microscopically, cerebellar atrophy was present in the form of widespread loss of Purkinje cells, with a shrunken appearance of rare residual Purkinje cells, mild cell loss in the granule cell layer and gliosis of the molecular layer.^21^, ^30^ Dentate nuclei, which are affected in multiple inherited SCAs,^77^, ^78^ showed no substantial atrophy in SCA27B.^21^ Furthermore, no patient exhibited intranuclear and cytoplasmic p62‐positive inclusions, polyglutamine immunoreactivity and cerebellar alpha‐synuclein or tau pathology.^21^, ^30^ Macroscopic and microscopic examination of the brainstem and spinal cord showed no apparent atrophy or neuronal cell loss in patients with SCA27B. In keeping with the elevated frequency of neurodegenerative diseases in elderly populations,^79^, ^80^, ^81^, ^82^ a concomitant independent pathological process was observed in two patients with SCA27B, namely diffuse neocortical Lewy body pathology and progressive supranuclear palsy pathology.^21^, ^30^

## 7 PROSPECTIVE TREATMENT STRATEGIES

Aside from omaveloxolone for Friedreich's ataxia,^83^ there currently is no other approved disease‐modifying therapy for SCAs.^84^ Acetazolamide and 4‐aminopyridine (4‐AP) have both been shown in randomised placebo‐controlled trials to similarly reduce the frequency of attacks in EA2.^76^, ^85^ In comparison, initial observations in patients with SCA27B suggested that 4‐AP, but not acetazolamide, may reduce the severity and frequency of ataxic symptoms.^21^ These initial findings were largely confirmed in subsequent open‐label series.^30^, ^31^, ^51^, ^52^ Specifically, a retrospective open‐label multicentre study found that only 44% of patients with SCA27B report a subjective mild symptomatic benefit with acetazolamide, which is sustained in 87% of them.^51^ In comparison, six of seven (86%) treated patients in one study reported a treatment response to 4‐AP of high effect size with relevance to everyday living.^30^ Prospective structured n‐of‐1 open‐label treatment experience in three patients further showed a tight on/off association between 4‐AP (10 mg extended release, twice daily) and symptomatic improvement of ataxia. Symptomatic time per day and days with severe symptoms both decreased while on 4‐AP. Furthermore, visual disturbances, vertigo and dysarthria were among the most responsive symptoms to 4‐AP.^30^ In a follow‐up prospective study of real‐life gait performance assessed by digital gait analysis, all evaluated gait parameters showed improvement while on the drug compared to off the drug (and no drug state, prior to initiation of treatment).^52^ In particular, stride variability and harmonic gait ratios improved while on the drugs,^52^ two parameters that have previously been shown to be associated with a higher risk of falls.^86^ This pilot study provided the first evidence that digital‐motor gait biomarkers may be sensitive, meaningful and ecologically valid outcome measures in future clinical trials, compared to the SARA score which failed to capture 4‐AP‐driven gait improvement.^52^ A recent study on a large cohort of patients with DBN provided further large‐scale evidence for the potential efficacy of 4‐AP in SCA27B.^31^ Patients with DBN carrying an *FGF14* expansion were found to have a significantly greater clinician‐reported (81 vs. 31%) and patient‐reported (59 vs. 11%) response rate to 4‐AP treatment (10 mg extended release, twice daily) compared to patients with DBN not carrying an expansion. This included, in some cases, a treatment response with high relevance to everyday living, as exemplified by an improvement of 2 FARS stages. Furthermore, re‐analysis of video‐oculography data of four patients – who were found in that study to carry an *FGF14* repeat expansion – from a previous randomised double‐blind, placebo‐controlled 4‐AP trial^87^ showed a significant improvement of slow phase velocity of the DBN with 4‐AP (5 mg immediate release, four times a day for 3 days followed by 10 mg immediate release, four times a day for 4 days), but not placebo.^31^ Together, these findings provide both large‐scale real‐world evidence as well as first preliminary placebo‐controlled evidence that 4‐AP may be effective in GAA‐*FGF14* disease/SCA27B. Nonetheless, a larger randomised placebo‐controlled trial will be needed to validate the promising benefits of 4‐AP in GAA‐*FGF14* disease/SCA27B reported thus far.

The potassium channel blocker 4‐AP may improve symptoms in SCA27B by restoring the rhythmic firing property of cerebellar Purkinje cells that is disrupted with the loss of *FGF14* function,^88^, ^89^ as shown in other forms of hereditary ataxia.^90^, ^91^ While the tight on/off association between 4‐AP intake and symptom improvement reported in recent studies suggests a symptomatic rather than disease‐modifying effect of 4‐AP in SCA27B,^30^, ^52^ a previous study in a mouse model of SCA1 has shown that early and chronic treatment with AP may have a neuroprotective effect through restoration of the firing rate of Purkinje cells.^92^ Whether early and chronic treatment with 4‐AP may slow down the neurodegenerative process in SCA27B will need further exploration.

## 8 MOLECULAR DIAGNOSIS

The diagnosis of SCA27B is established in a symptomatic individual with characteristic clinical findings by the identification of a heterozygous GAA repeat expansion in the first intron of *FGF14*.^21^, ^22^ Affected individuals usually carry 250 or more GAA repeats, although recent data have raised the possibility of a lower pathogenic threshold (Figure 1A).^23^, ^31^ Ongoing multicentre screening efforts are currently exploring the lower boundaries of the pathogenic threshold and the incompletely penetrant range. Since a substantial percentage of cases are sporadic (15–50%),^21^, ^30^, ^41^, ^50^ patients with a compatible phenotype should be screened for SCA27B regardless of family history. As of today, since short‐read sequencing cannot accurately size *FGF14* GAA expansions,^22^ molecular testing for SCA27B relies on a bespoke targeted PCR‐based gene analysis to determine the length and purity of *FGF14* GAA repeats.^41^ With accumulating evidence suggesting that non‐GAA‐pure repeat expansions are likely non‐pathogenic for ataxia,^21^, ^23^, ^34^ it becomes imperative to adopt standardised diagnostic strategies to diagnose SCA27B. One such strategy, which was found to compare favourably to long‐read sequencing and subsequently validated in a French cohort, relies on the combination of fragment length analysis of fluorescent long‐range PCR amplification products, bidirectional repeat‐primed PCRs, and, in some cases, agarose gel electrophoresis of long‐range PCR amplification products and/or Sanger sequencing.^41^ A comprehensive assessment of the repeat locus is necessary given its high degree of length and sequence polymorphism in the general population.^32^ In addition to relying on the identification of GAA‐pure repeat expansions, the diagnosis of SCA27B must also be made in patients with a compatible phenotype. This is of particular importance given the incomplete penetrance of (GAA)_250‐300_ expansions,^21^, ^22^ which may not be pathogenic in individuals whose phenotype differs significantly from SCA27B. The relatively elevated population allele frequency of *FGF14* repeat expansions^21^, ^22^, ^32^ increases the likelihood that a co‐morbid neurodegenerative disease or a second pathogenic variant in another ataxia gene co‐occur in a single patient, thus complexifying the underlying phenotype (which should not be interpreted as being indicative of a broader phenotypic spectrum of SCA27B).^30^

Short‐read sequencing approaches are capable of showing whether an individual has an *FGF14* GAA repeat longer than approximately 50 repeat units.^93^ That can be useful for screening purposes if individuals already have short‐read WGS data available as over 80% of alleles at this locus are shorter than 50 repeat units.^32^ However, short‐read WGS is incapable of accurately sizing the allele beyond that length and is also not able to accurately detect motif impurity for longer alleles. As such, short‐read WGS must always be followed by suitable techniques such as long‐range PCR and repeat‐primed PCR in order to establish a diagnosis of SCA27B.

Long‐read sequencing approaches such as PacBio HiFi and Oxford Nanopore hold promise as single assays that could eventually be used to diagnose individuals with SCA27B, but currently each method is hampered by some drawbacks. PacBio HiFi reads often struggle to generate consensus sequences on long GAA‐pure tracts,^94^ such as those in SCA27B patients, which can lead to allele dropout and false negative diagnoses. Oxford Nanopore reads may generate systematic errors in basecalling which can lead to erroneous identification of GAA impurities in SCA27B patients.^95^ This can either lead to false negative diagnoses or false positive diagnoses if the GAA purity threshold is decreased for this testing modality.

## 9 PATHOGENIC MECHANISMS IN SCA27B

*FGF14* encodes the intracellular fibroblast growth factor 14 protein that is widely expressed throughout the central nervous system, most abundantly in the cerebellum.^27^ FGF14 regulates spontaneous and evoked firing of Purkinje cells by interacting with and modulating the function of voltage‐gated sodium channels at the axon initial segment.^96^, ^97^, ^98^ Loss of FGF14 function in mice has been shown to attenuate repetitive firing of Purkinje cells as a result of impairment of sodium channel kinetics, ultimately leading to motor incoordination and imbalance.^88^, ^89^, ^99^

Since SCA27B is caused by expansion of GAA tandem repeats, it is possible that its pathomechanism shares common features with Friedreich ataxia (FRDA). In FRDA, GAA triplets expanded beyond 66 repeat units form a DNA secondary structure termed ‘sticky DNA’, which inhibits transcription of the *FXN* gene.^100^, ^101^, ^102^ Since RNA and protein expression of *FGF14* was found to be decreased in post‐mortem cerebellum samples and induced pluripotent stem cell‐derived motor neurons of SCA27B patients,^21^ the current evidence suggests that SCA27B is caused by loss of function of *FGF14*, potentially through inhibition of transcription by the sticky DNA secondary structure. Further observations from FRDA that need to be tested to determine if they also exist in SCA27B include the DNA hypermethylation of the expanded repeat, heterochromatin formation and additional epigenetic changes, which also drive the transcription inhibition in FRDA.^103^, ^104^, ^105^, ^106^

## 10 THE ATAXIA GLOBAL INITIATIVE: BRINGING RESEARCH ON SCA27B TO A GLOBAL SCALE

**FIGURE 2:**
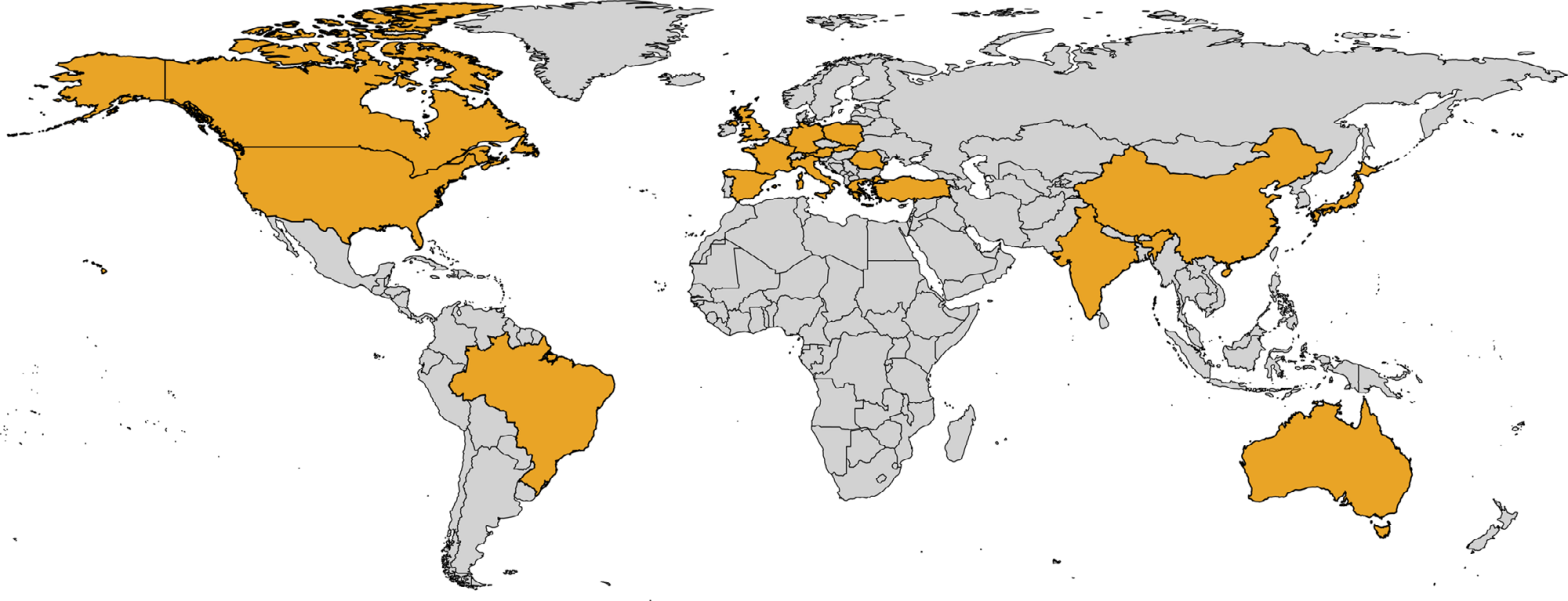
Geographic distribution of identified patients with spinocerebellar ataxia 27B. Patients with spinocerebellar ataxia 27B (SCA27B) have now been identified in 18 countries around the world, shown in orange in the world map.

The Ataxia Global Initiative (AGI) (https://ataxia‐global‐initiative.net) is a worldwide research platform established in 2021 that brings together academic and industry partners and patient organisations to facilitate the clinical development of therapies for ataxias.^14^ The AGI promotes the coordination and facilitation of collaboration in ataxia research toward achieving trial readiness by aggregating cohorts and standardising biomarkers and outcomes measures.^14^ Leveraging this unique platform, an international AGI‐endorsed SCA27B Study Group was launched in 2023 in response to immediate need to set the stage for trial readiness in SCA27B given its apparently high worldwide prevalence and the early promising treatment results with 4‐AP. This international project, which now includes over 50 participating clinical centres worldwide, will allow for: (i) adoption of a standardised testing strategy to diagnose SCA27B; (ii) defining the frequency of SCA27B in different populations; (iii) delineation of the phenotypic profile of SCA27B through real‐world registry data capture; (iv) establishing the relationship between age at disease onset and the size of the expansion, and the age‐dependent penetrance of the disease; and (v) reaching a consensus on the outcome measures to use in future trials. More than 500 cases of SCA27B from 18 countries around the world have so far been identified through this collaboration (Figure 2). Such a global collaborative effort is expected to accelerate research in SCA27B, thereby setting the stage for trial readiness.

## 11 CONCLUSION

In this review, we have discussed what is currently known about SCA27B from molecular, genetic, epidemiological and clinical perspectives. Though the genetic cause of this disease was discovered very recently, evidence has accumulated rapidly that it is one of the most common causes of adult‐onset ataxia. It is fortunate that a potentially effective symptomatic treatment emerged quickly for SCA27B in the form of 4‐AP, but it is possible that small molecules designed to treat FRDA such as Syn‐TEF1 (now in phase I trial; see https://www.curefa.org/research/research‐pipeline), which binds to expanded GAA repeats to enable transcription,^107^ could ultimately prove to have a beneficial disease‐modifying effect in patients with SCA27B. Looking ahead, there is much work to be done but also tremendous enthusiasm to accurately diagnose affected individuals, understand the pathomechanism and ultimately identify effective treatment options for SCA27B.

## AUTHOR CONTRIBUTION

All authors contributed to background research, discussion of the content and reviewed and/or edited the manuscript.

## STUDY SPONSORSHIP AND FUNDING

This work was supported by the Clinician Scientist program ‘PRECISE.net’ funded by the Else Kröner‐Fresenius‐Stiftung (to M. S.) and the grant 779257 ‘Solve‐RD’ from the European's Union Horizon 2020 research and innovation program (to M. S. and H. H.). This work was also supported by the Deutsche Forschungsgemeinschaft (DFG, German Research Foundation) N° 441409627, as part of the PROSPAX consortium under the frame of EJP RD, the European Joint Programme on Rare Diseases, under the EJP RD COFUND‐EJP N° 825575 (to M. S., B. B. and – as associated partner – S. Z.), the NIH National Institute of Neurological Disorders and Stroke (grant 2R01NS072248‐11A1 to S. Z.) and the Fondation Groupe Monaco (to B. B.). H. H. is supported by the Wellcome Trust, the UK Medical Research Council (MRC) and by the UCLH/UCL Biomedical Research Centre. The funders had no role in the conduct of this study.

## CONFLICT OF INTEREST STATEMENT

D. P., M. C. D., M. R., H. H. and B. B. report no disclosures. M. S. has received consultancy honoraria from Janssen, Ionis, Orphazyme, Servier, Reata, Biohaven, Zevra, Lilly, GenOrph and AviadoBio, all unrelated to the present manuscript. M. S. is planning a treatment trial of 4‐AP in GAA‐*FGF14* disease together with Solaxa Inc. as a sponsor, but has not received any type of honoraria or funding from Solaxa. S. Z. has received consultancy honoraria from Neurogene, Aeglea BioTherapeutics, Applied Therapeutics and is an unpaid officer of the TGP foundation, all unrelated to the present manuscript.

## ETHICS STATEMENT

Not applicable.

## References

[1] Smedley D , Smith KR , Martin A , et al. 100,000 Genomes pilot on rare‐disease diagnosis in health care—preliminary report. N Engl J Med. 2021;385:1868‐1880. 34758253 10.1056/NEJMoa2035790 PMC7613219

[2] Boycott KM , Rath A , Chong JX , et al. International cooperation to enable the diagnosis of all rare genetic diseases. Am J Hum Genet. 2017;100:695‐705. 28475856 10.1016/j.ajhg.2017.04.003 PMC5420351

[3] Rexach J , Lee H , Martinez‐Agosto JA , Németh AH , Fogel BL . Clinical application of next‐generation sequencing to the practice of neurology. Lancet Neurol. 2019;18:492‐503. 30981321 10.1016/S1474-4422(19)30033-X PMC7055532

[4] Schuermans N , Verdin H , Ghijsels J , et al. Exome sequencing and multigene panel testing in 1,411 patients with adult‐onset neurologic disorders. Neurol Genet. 2023;9:e200071. 37152446 10.1212/NXG.0000000000200071 PMC10160959

[5] Perlman S . Hereditary Ataxia Overview. In: Adam MP , Mirzaa GM , Pagon RA , Wallace SE , Bean LJH , Gripp KW , Amemiya A , eds. GeneReviews(®).University of Washington, Seattle; 1993. 20301317

[6] Ngo KJ , Rexach JE , Lee H , et al. A diagnostic ceiling for exome sequencing in cerebellar ataxia and related neurological disorders. Hum Mutat. 2020;41:487‐501. 31692161 10.1002/humu.23946 PMC7182470

[7] Galatolo D , Tessa A , Filla A , Santorelli FM . Clinical application of next generation sequencing in hereditary spinocerebellar ataxia: increasing the diagnostic yield and broadening the ataxia‐spasticity spectrum. A retrospective analysis. Neurogenetics. 2018;19:1‐8. 29209898 10.1007/s10048-017-0532-6

[8] Giordano I , Harmuth F , Jacobi H , et al. Clinical and genetic characteristics of sporadic adult‐onset degenerative ataxia. Neurology. 2017;89:1043‐1049. 28794257 10.1212/WNL.0000000000004311

[9] Bogdan T , Wirth T , Iosif A , et al. Unravelling the etiology of sporadic late‐onset cerebellar ataxia in a cohort of 205 patients: a prospective study. J Neurol. 2022;269:6354‐6365. 35869996 10.1007/s00415-022-11253-1

[10] da Graça FF , Peluzzo TM , Bonadia LC , et al. Diagnostic yield of whole exome sequencing for adults with ataxia: a Brazilian perspective. Cerebellum. 2022;21:49‐54. 33956305 10.1007/s12311-021-01268-1

[11] Mahmoud M , Gobet N , Cruz‐Dávalos DI , Mounier N , Dessimoz C , Sedlazeck FJ . Structural variant calling: the long and the short of it. Genome Biol. 2019;20:246. 31747936 10.1186/s13059-019-1828-7 PMC6868818

[12] Bahlo M , Bennett MF , Degorski P , Tankard RM , Delatycki MB , Lockhart PJ . Recent advances in the detection of repeat expansions with short‐read next‐generation sequencing. F1000Research. 2018;7:F1000Faculty Rev‐736. 10.12688/f1000research.13980.1 PMC6008857 29946432

[13] Depienne C , Mandel JL . 30 years of repeat expansion disorders: what have we learned and what are the remaining challenges? Am J Hum Genet. 2021;108:764‐785. 33811808 10.1016/j.ajhg.2021.03.011 PMC8205997

[14] Klockgether T , Ashizawa T , Brais B , et al. Paving the way toward meaningful trials in ataxias: an ataxia global initiative perspective. Mov Disord. 2022;37:1125‐1130. 35475582 10.1002/mds.29032

[15] Vázquez‐Mojena Y , León‐Arcia K , González‐Zaldivar Y , Rodríguez‐Labrada R , Velázquez‐Pérez L . Gene therapy for polyglutamine spinocerebellar ataxias: advances, challenges, and perspectives. Mov Disord. 2021;36:2731‐2744. 34628681 10.1002/mds.28819

[16] Synofzik M , Puccio H , Mochel F , Schöls L . Autosomal recessive cerebellar ataxias: paving the way toward targeted molecular therapies. Neuron. 2019;101:560‐583. 30790538 10.1016/j.neuron.2019.01.049

[17] Mantere T , Kersten S , Hoischen A . Long‐read sequencing emerging in medical genetics. Front Genet. 2019;10:426. 31134132 10.3389/fgene.2019.00426 PMC6514244

[18] Su Y , Fan L , Shi C , et al. Deciphering neurodegenerative diseases using long‐read sequencing. Neurology. 2021;97:423‐433. 34389649 10.1212/WNL.0000000000012466 PMC8408508

[19] Cortese A , Simone R , Sullivan R , et al. Biallelic expansion of an intronic repeat in RFC1 is a common cause of late‐onset ataxia. Nat Genet. 2019;51:649‐658. 30926972 10.1038/s41588-019-0372-4 PMC6709527

[20] Rafehi H , Szmulewicz DJ , Bennett MF , et al. Bioinformatics‐based identification of expanded repeats: a non‐reference intronic pentamer expansion in RFC1 causes CANVAS. Am J Hum Genet. 2019;105:151‐165. 31230722 10.1016/j.ajhg.2019.05.016 PMC6612533

[21] Pellerin D , Danzi MC , Wilke C , et al. Deep intronic FGF14 GAA repeat expansion in late‐onset cerebellar ataxia. N Engl J Med. 2023;388:128‐141. 36516086 10.1056/NEJMoa2207406 PMC10042577

[22] Rafehi H , Read J , Szmulewicz DJ , et al. An intronic GAA repeat expansion in FGF14 causes the autosomal‐dominant adult‐onset ataxia SCA27B/ATX‐FGF14. Am J Hum Genet. 2023;110:1018. 37267898 10.1016/j.ajhg.2023.05.005 PMC10257192

[23] Hengel H , Pellerin D , Wilke C , et al. As frequent as polyglutamine spinocerebellar ataxias: sCA27B in a large german autosomal dominant ataxia cohort. Mov Disord. 2023;38:1557‐1558. 37528564 10.1002/mds.29559

[24] van Swieten JC , Brusse E , de Graaf BM , et al. A mutation in the fibroblast growth factor 14 gene is associated with autosomal dominant cerebellar ataxia [corrected]. Am J Hum Genet. 2003;72:191‐199. 12489043 10.1086/345488 PMC378625

[25] Dalski A , Atici J , Kreuz FR , Hellenbroich Y , Schwinger E , Zühlke C . Mutation analysis in the fibroblast growth factor 14 gene: frameshift mutation and polymorphisms in patients with inherited ataxias. Eur J Hum Genet. 2005;13:118‐120. 15470364 10.1038/sj.ejhg.5201286

[26] Piarroux J , Riant F , Humbertclaude V , et al. FGF14‐related episodic ataxia: delineating the phenotype of episodic ataxia type 9. Ann Clin Transl Neurol. 2020;7:565‐572. 32162847 10.1002/acn3.51005 PMC7187715

[27] Wang Q , Bardgett ME , Wong M , et al. Ataxia and paroxysmal dyskinesia in mice lacking axonally transported FGF14. Neuron. 2002;35:25‐38. 12123606 10.1016/s0896-6273(02)00744-4

[28] Brais B , Pellerin D , Danzi MC . Deep intronic FGF14 GAA repeat expansion in late‐onset cerebellar ataxia. Reply. N Engl J Med. 2023;388:e70. 10.1056/NEJMc2301605 37224216

[29] Zeng YH , Gan SR , Chen WJ . Deep intronic FGF14 GAA repeat expansion in late‐onset cerebellar ataxia. N Engl J Med. 2023;388:e70. 10.1056/NEJMc2301605 37224214

[30] Wilke C , Pellerin D , Mengel D , et al. GAA‐FGF14 ataxia (SCA27B): phenotypic profile, natural history progression and 4‐aminopyridine treatment response. Brain. 2023;146:4144‐4157. 37165652 10.1093/brain/awad157

[31] Pellerin D , Heindl F , Wilke C , et al. Intronic *FGF14* GAA repeat expansions are a common cause of downbeat nystagmus syndromes: frequency, phenotypic profile, and 4‐aminopyridine treatment response. medRxiv. 2023. 2023.2007.2030.23293380.

[32] Pellerin D , Gobbo GD , Couse M , et al. A common flanking variant is associated with enhanced meiotic stability of the *FGF14*‐SCA27B locus. Biorxiv. 2023. 2023.2005.2011.540430.

[33] Saffie Awad P , Lohmann K , Hirmas Y , et al. Shaking up ataxia: fGF14 and RFC1 repeat expansions in affected and unaffected members of a Chilean family. Mov Disord. 2023;38:1107‐1109. 37246629 10.1002/mds.29390

[34] Pellerin D , Iruzubieta P , Tekgül Ş , et al. Non‐GAA repeat expansions in FGF14 are likely not pathogenic‐reply to: “Shaking up ataxia: fGF14 and RFC1 repeat expansions in affected and unaffected members of a chilean family”. Mov Disord. 2023;38:1575‐1577. 37565404 10.1002/mds.29552

[35] Iruzubieta P , Pellerin D , Bergareche A , et al. Frequency and phenotypic spectrum of spinocerebellar ataxia 27B and other genetic ataxias in a Spanish cohort of late‐onset cerebellar ataxia. Eur J Neurol. 2023;30:3828‐3833. 37578187 10.1111/ene.16039

[36] Ruano L , Melo C , Silva MC , Coutinho P . The global epidemiology of hereditary ataxia and spastic paraplegia: a systematic review of prevalence studies. Neuroepidemiology. 2014;42:174‐183. 24603320 10.1159/000358801

[37] Muzaimi MB , Thomas J , Palmer‐Smith S , et al. Population based study of late onset cerebellar ataxia in south east Wales. J Neurol Neurosurg Psychiatry. 2004;75:1129‐1134. 15258214 10.1136/jnnp.2003.014662 PMC1739172

[38] Leone M , Bottacchi E , D'Alessandro G , Kustermann S . Hereditary ataxias and paraplegias in Valle d'Aosta, Italy: a study of prevalence and disability. Acta Neurol Scand. 1995;91:183‐187. 7793232 10.1111/j.1600-0404.1995.tb00430.x

[39] Polo JM , Calleja J , Combarros O , Berciano J . Hereditary ataxias and paraplegias in Cantabria, Spain. An epidemiological and clinical study. Brain. 1991;114(2):855‐866. Pt. 2043954 10.1093/brain/114.2.855

[40] Tsuji S , Onodera O , Goto J , Nishizawa M . Sporadic ataxias in Japan–a population‐based epidemiological study. Cerebellum. 2008;7:189‐197. 18418674 10.1007/s12311-008-0028-x

[41] Bonnet C , Pellerin D , Roth V , et al. Optimized testing strategy for the diagnosis of GAA‐FGF14 ataxia/spinocerebellar ataxia 27B. Sci Rep. 2023;13:9737. 37322040 10.1038/s41598-023-36654-8 PMC10272173

[42] Wirth T , Clément G , Delvallée C , et al. Natural history and phenotypic spectrum of GAA‐FGF14 sporadic late‐onset cerebellar ataxia (SCA27B). Mov Disord. 2023;38:1950‐1956. 37470282 10.1002/mds.29560

[43] Novis LE , Frezatti RS , Pellerin D , et al. Frequency of GAA‐*FGF14* ataxia in a large cohort of Brazilian patients with unsolved adult‐onset cerebellar ataxia. Neurology Genetics. 2023;9:e2000094. 10.1212/NXG.0000000000200094 PMC10461713 37646005

[44] Mizushima K , Shibata Y , Shirai S , Matsushima, M , Miyatake, S , Iwata, I , Yaguchi, H , Matsumoto, N , & Yabe, I Prevalence of repeat expansions causing autosomal dominant spinocerebellar ataxias in Hokkaido, the northernmost island of Japan. Journal of human genetics. 2023. doi:10.1038/s10038-023-01200-x 37848721

[45] Ando M , Higuchi Y , Yuan J , et al. Clinical variability associated with intronic FGF14 GAA repeat expansion in Japan. Ann Clin Transl Neurol. 2023. doi:10.1002/acn3.51936 PMC10791012 37916889

[46] Alshimemeri S , Abo Alsamh D , Zhou L , et al. Demographics and clinical characteristics of autosomal dominant spinocerebellar ataxia in Canada. Mov Disord Clin Pract. 2023;10:440‐451. 36949783 10.1002/mdc3.13666 PMC10026276

[47] Scriver CR . Human genetics: lessons from Quebec populations. Annu Rev Genomics Hum Genet. 2001;2:69‐101. 11701644 10.1146/annurev.genom.2.1.69

[48] Jacobi H , du Montcel ST , Bauer P , et al. Long‐term disease progression in spinocerebellar ataxia types 1, 2, 3, and 6: a longitudinal cohort study. Lancet Neurol. 2015;14:1101‐1108. 26377379 10.1016/S1474-4422(15)00202-1

[49] Tezenas du Montcel S , Durr A , Rakowicz M , et al. Prediction of the age at onset in spinocerebellar ataxia type 1, 2, 3 and 6. J Med Genet. 2014;51:479‐486. 24780882 10.1136/jmedgenet-2013-102200 PMC4078703

[50] Ashton C , Indelicato E , Pellerin D , Clément, G , Danzi, MC , Dicaire, MJ , Bonnet, C , Houlden, H. , Züchner, S , Synofzik, M , Lamont, PJ , Renaud, M , Boesch, S , & Brais, B . Spinocerebellar ataxia 27B: episodic symptoms and acetazolamide response in 34 patients. Brain communications. 2023:5(5), fcad239. 10.1093/braincomms/fcad239 PMC10495284 37705681

[51] Ashton C , Indelicato E , Pellerin D , et al. Spinocerebellar ataxia 27B: episodic symptoms and acetazolamide response in 34 patients. Brain Commun. 2023;5(5):fcad239. 10.1093/braincomms/fcad239 37705681 PMC10495284

[52] Seemann J , Traschütz A , Ilg W , Synofzik M . 4‐Aminopyridine improves real‐life gait performance in SCA27B on a single‐subject level: a prospective n‐of‐1 treatment experience. J Neurol. 2023;270:5629‐5634. 37439944 10.1007/s00415-023-11868-y PMC10576659

[53] Yabe I , Sasaki H , Takeichi N , et al. Positional vertigo and macroscopic downbeat positioning nystagmus in spinocerebellar ataxia type 6 (SCA6). J Neurol. 2003;250:440‐443. 12700909 10.1007/s00415-003-1020-5

[54] Globas C , du Montcel ST , Baliko L , et al. Early symptoms in spinocerebellar ataxia type 1, 2, 3, and 6. Mov Disord. 2008;23:2232‐2238. 18759344 10.1002/mds.22288

[55] Borsche M , Thomsen M , Szmulewicz DJ , et al. Bilateral vestibulopathy in RFC1‐positive CANVAS is distinctly different compared to FGF14‐linked spinocerebellar ataxia 27B. J Neurol. 2023. doi:10.1007/s00415-023-12050-0 PMC10827886 37861706

[56] Currò R , Salvalaggio A , Tozza S , et al. RFC1 expansions are a common cause of idiopathic sensory neuropathy. Brain. 2021;144:1542‐1550. 33969391 10.1093/brain/awab072 PMC8262986

[57] Huin V , Coarelli G , Guemy C , et al. Motor neuron pathology in CANVAS due to RFC1 expansions. Brain. 2022;145:2121‐2132. 34927205 10.1093/brain/awab449

[58] Traschütz A , Cortese A , Reich S , et al. Natural history, phenotypic spectrum, and discriminative features of multisystemic RFC1 disease. Neurology. 2021;96:e1369‐e1382. 33495376 10.1212/WNL.0000000000011528 PMC8055326

[59] Hadjivassiliou M , Currò R , Beauchamp N , Dominik, N , Grunewald, RA , Shanmugarajah, P , Zis, P , Hoggard, N , & Cortese, A . Can CANVAS due to RFC1 biallelic expansions present with pure ataxia? Journal of neurology, neurosurgery, and psychiatry. 2023:jnnp‐2023‐331381. Advance online publication. 10.1136/jnnp-2023-331381 PMC10850715 37414537

[60] Synofzik M , Schüle R . Overcoming the divide between ataxias and spastic paraplegias: shared phenotypes, genes, and pathways. Mov Disord. 2017;32:332‐345. 28195350 10.1002/mds.26944 PMC6287914

[61] Coarelli G , Wirth T , Tranchant C , Koenig M , Durr A , Anheim M . The inherited cerebellar ataxias: an update. J Neurol. 2023;270:208‐222. 36152050 10.1007/s00415-022-11383-6 PMC9510384

[62] Poewe W , Stankovic I , Halliday G , et al. Multiple system atrophy. Nat Rev Dis Primers. 2022;8:56. 36008429 10.1038/s41572-022-00382-6

[63] Fanciulli A , Wenning GK . Multiple‐system atrophy. N Engl J Med. 2015;372:249‐263. 25587949 10.1056/NEJMra1311488

[64] Wenning GK , Stankovic I , Vignatelli L , et al. The movement disorder society criteria for the diagnosis of multiple system atrophy. Mov Disord. 2022;37:1131‐1148. 35445419 10.1002/mds.29005 PMC9321158

[65] Casey HL , Gomez CM . Spinocerebellar ataxia type 6. In: Adam MP , Feldman J , Mirzaa GM , eds. GeneReviews(®).University of Washington, Seattle. 20301319

[66] Copyright © 1993–2023, University of Washington, Seattle. GeneReviews is a registered trademark of the University of Washington, Seattle. All rights reserved., 1993.

[67] Takahashi H , Ishikawa K , Tsutsumi T , et al. A clinical and genetic study in a large cohort of patients with spinocerebellar ataxia type 6. J Hum Genet. 2004;49:256‐264. 15362569 10.1007/s10038-004-0142-7

[68] Gomez CM , Thompson RM , Gammack JT , et al. Spinocerebellar ataxia type 6: gaze‐evoked and vertical nystagmus, Purkinje cell degeneration, and variable age of onset. Ann Neurol. 1997;42:933‐950. 9403487 10.1002/ana.410420616

[69] Shirai S , Mizushima K , Fujiwara K , et al. Case series: downbeat nystagmus in SCA27B. J Neurol Sci. 2023;454:120849. 37907039 10.1016/j.jns.2023.120849

[70] Butteriss D , Chinnery P , Birchall D . Radiological characterization of spinocerebellar ataxia type 6. Br J Radiol. 2005;78:694‐696. 16046419 10.1259/bjr/73834093

[71] Imbrici P , Eunson LH , Graves TD , et al. Late‐onset episodic ataxia type 2 due to an in‐frame insertion in CACNA1A. Neurology. 2005;65:944‐946. 16186543 10.1212/01.wnl.0000176069.64200.28

[72] Baloh RW . Episodic ataxias 1 and 2. Handb Clin Neurol. 2012;103:595‐602. 21827920 10.1016/B978-0-444-51892-7.00042-5

[73] Nachbauer W , Nocker M , Karner E , et al. Episodic ataxia type 2: phenotype characteristics of a novel CACNA1A mutation and review of the literature. J Neurol. 2014;261:983‐991. 24658662 10.1007/s00415-014-7310-2

[74] Jen JC , Wan J . Episodic ataxias. Handb Clin Neurol. 2018;155:205‐215. 29891059 10.1016/B978-0-444-64189-2.00013-5

[75] Jen J , Kim GW , Baloh RW . Clinical spectrum of episodic ataxia type 2. Neurology. 2004;62:17‐22. 14718690 10.1212/01.wnl.0000101675.61074.50

[76] Hassan A . Episodic ataxias: primary and secondary etiologies, treatment, and classification approaches. Tremor Other Hyperkinet Mov (N Y). 2023;13:9. 37008993 10.5334/tohm.747 PMC10064912

[77] Muth C , Teufel J , Schöls L , et al. Fampridine and acetazolamide in EA2 and related familial EA: a prospective randomized placebo‐controlled trial. Neurol Clin Pract. 2021;11:e438‐e446. 34484942 10.1212/CPJ.0000000000001017 PMC8382428

[78] Koeppen AH . The pathogenesis of spinocerebellar ataxia. Cerebellum. 2005;4:62‐73. 15895563 10.1080/14734220510007950

[79] Deistung A , Jäschke D , Draganova R , et al. Quantitative susceptibility mapping reveals alterations of dentate nuclei in common types of degenerative cerebellar ataxias. Brain Commun. 2022;4:fcab306. 35291442 10.1093/braincomms/fcab306 PMC8914888

[80] Sonnen JA , Santa Cruz K , Hemmy LS , et al. Ecology of the aging human brain. Arch Neurol. 2011;68:1049‐1056. 21825242 10.1001/archneurol.2011.157 PMC3218566

[81] Beach TG , Malek‐Ahmadi M . Alzheimer's disease neuropathological comorbidities are common in the younger‐old. J Alzheimer's Dis. 2021;79:389‐400. 33285640 10.3233/JAD-201213 PMC8034496

[82] Brenowitz WD , Keene CD , Hawes SE , et al. Alzheimer's disease neuropathologic change, Lewy body disease, and vascular brain injury in clinic‐ and community‐based samples. Neurobiol Aging. 2017;53:83‐92. 28236716 10.1016/j.neurobiolaging.2017.01.017 PMC5385292

[83] Hou Y , Dan X , Babbar M , et al. Ageing as a risk factor for neurodegenerative disease. Nat Rev Neurol. 2019;15:565‐581. 31501588 10.1038/s41582-019-0244-7

[84] Mullard A . 2023:22(4)258. FDA approves first Friedreich's ataxia drug. Nature reviews Drug discovery. 10.1038/d41573-023-00041-9 36890218

[85] Zesiewicz TA , Wilmot G , Kuo SH , et al. Comprehensive systematic review summary: treatment of cerebellar motor dysfunction and ataxia: report of the Guideline Development, Dissemination, and Implementation Subcommittee of the American Academy of Neurology. Neurology. 2018;90:464‐471. 29440566 10.1212/WNL.0000000000005055 PMC5863491

[86] Strupp M , Kalla R , Claassen J , et al. A randomized trial of 4‐aminopyridine in EA2 and related familial episodic ataxias. Neurology. 2011;77:269‐275. 21734179 10.1212/WNL.0b013e318225ab07 PMC3136055

[87] Castiglia SF , Trabassi D , Tatarelli A , et al. Identification of gait unbalance and fallers among subjects with cerebellar ataxia by a set of trunk acceleration‐derived indices of gait. Cerebellum. 2023;22:46‐58. 35079958 10.1007/s12311-021-01361-5

[88] Claassen J , Spiegel R , Kalla R , et al. A randomised double‐blind, cross‐over trial of 4‐aminopyridine for downbeat nystagmus–effects on slowphase eye velocity, postural stability, locomotion and symptoms. J Neurol Neurosurg Psychiatry. 2013;84:1392‐1399. 23813743 10.1136/jnnp-2012-304736

[89] Bosch MK , Carrasquillo Y , Ransdell JL , Kanakamedala A , Ornitz DM , Nerbonne JM . Intracellular FGF14 (iFGF14) is required for spontaneous and evoked firing in cerebellar purkinje neurons and for motor coordination and balance. J Neurosci. 2015;35:6752‐6769. 25926453 10.1523/JNEUROSCI.2663-14.2015 PMC4412895

[90] Shakkottai VG , Xiao M , Xu L , et al. FGF14 regulates the intrinsic excitability of cerebellar Purkinje neurons. Neurobiol Dis. 2009;33:81‐88. 18930825 10.1016/j.nbd.2008.09.019 PMC2652849

[91] Alviña K , Khodakhah K . The therapeutic mode of action of 4‐aminopyridine in cerebellar ataxia. J Neurosci. 2010;30:7258‐7268. 20505092 10.1523/JNEUROSCI.3582-09.2010 PMC2909847

[92] Jayabal S , Chang HH , Cullen KE , Watt AJ . 4‐aminopyridine reverses ataxia and cerebellar firing deficiency in a mouse model of spinocerebellar ataxia type 6. Sci Rep. 2016;6:29489. 27381005 10.1038/srep29489 PMC4933933

[93] Hourez R , Servais L , Orduz D , et al. Aminopyridines correct early dysfunction and delay neurodegeneration in a mouse model of spinocerebellar ataxia type 1. J Neurosci. 2011;31:11795‐11807. 21849540 10.1523/JNEUROSCI.0905-11.2011 PMC6623197

[94] Dolzhenko E , van Vugt J , Shaw RJ , et al. Detection of long repeat expansions from PCR‐free whole‐genome sequence data. Genome Res. 2017;27:1895‐1903. 28887402 10.1101/gr.225672.117 PMC5668946

[95] Rabanal FA , Gräff M , Lanz C , et al. Pushing the limits of HiFi assemblies reveals centromere diversity between two Arabidopsis thaliana genomes. Nucleic Acids Res. 2022;50:12309‐12327. 36453992 10.1093/nar/gkac1115 PMC9757041

[96] Krishnakumar R , Sinha A , Bird SW , et al. Systematic and stochastic influences on the performance of the MinION nanopore sequencer across a range of nucleotide bias. Sci Rep. 2018;8:3159. 29453452 10.1038/s41598-018-21484-w PMC5816649

[97] Xiao M , Bosch MK , Nerbonne JM , Ornitz DM . FGF14 localization and organization of the axon initial segment. Mol Cell Neurosci. 2013;56:393‐403. 23891806 10.1016/j.mcn.2013.07.008 PMC3791165

[98] Di Re J , Wadsworth PA , Laezza F . Intracellular fibroblast growth factor 14: emerging risk factor for brain disorders. Front Cell Neurosci. 2017;11:103. 28469558 10.3389/fncel.2017.00103 PMC5396478

[99] Lou JY , Laezza F , Gerber BR , et al. Fibroblast growth factor 14 is an intracellular modulator of voltage‐gated sodium channels. J Physiol. 2005;569:179‐193. 16166153 10.1113/jphysiol.2005.097220 PMC1464207

[100] Yan H , Pablo JL , Wang C , Pitt GS . FGF14 modulates resurgent sodium current in mouse cerebellar Purkinje neurons. eLife. 2014;3:e04193. 25269146 10.7554/eLife.04193 PMC4356139

[101] Sakamoto N , Chastain PD , Parniewski P , et al. Sticky DNA: self‐association properties of long GAA.TTC repeats in R.R.Y triplex structures from Friedreich's ataxia. Mol Cell. 1999;3:465‐475. 10230399 10.1016/s1097-2765(00)80474-8

[102] Sakamoto N , Larson JE , Iyer RR , Montermini L , Pandolfo M , Wells RD . GGA*TCC‐interrupted triplets in long GAA*TTC repeats inhibit the formation of triplex and sticky DNA structures, alleviate transcription inhibition, and reduce genetic instabilities. J Biol Chem. 2001;276:27178‐27187. 11325966 10.1074/jbc.M101852200

[103] Ohshima K , Montermini L , Wells RD , Pandolfo M . Inhibitory effects of expanded GAA.TTC triplet repeats from intron I of the Friedreich ataxia gene on transcription and replication in vivo. J Biol Chem. 1998;273:14588‐14595. 9603975 10.1074/jbc.273.23.14588

[104] Al‐Mahdawi S , Pinto RM , Ismail O , et al. The Friedreich ataxia GAA repeat expansion mutation induces comparable epigenetic changes in human and transgenic mouse brain and heart tissues. Hum Mol Genet. 2008;17:735‐746. 18045775 10.1093/hmg/ddm346

[105] Soragni E , Herman D , Dent SY , Gottesfeld JM , Wells RD , Napierala M . Long intronic GAA*TTC repeats induce epigenetic changes and reporter gene silencing in a molecular model of Friedreich ataxia. Nucleic Acids Res. 2008;36:6056‐6065. 18820300 10.1093/nar/gkn604 PMC2577344

[106] Greene E , Mahishi L , Entezam A , Kumari D , Usdin K . Repeat‐induced epigenetic changes in intron 1 of the frataxin gene and its consequences in Friedreich ataxia. Nucleic Acids Res. 2007;35:3383‐3390. 17478498 10.1093/nar/gkm271 PMC1904289

[107] Saveliev A , Everett C , Sharpe T , Webster Z , Festenstein R . DNA triplet repeats mediate heterochromatin‐protein‐1‐sensitive variegated gene silencing. Nature. 2003;422:909‐913. 12712207 10.1038/nature01596

[108] Erwin GS , Grieshop MP , Ali A , et al. Synthetic transcription elongation factors license transcription across repressive chromatin. Science. 2017;358:1617‐1622. 29192133 10.1126/science.aan6414 PMC6037176

